# Measuring the costs of biosecurity on poultry farms: a case study in broiler production in Finland

**DOI:** 10.1186/1751-0147-54-12

**Published:** 2012-02-28

**Authors:** Kirsi-Maarit Siekkinen, Jaakko Heikkilä, Niina Tammiranta, Heidi Rosengren

**Affiliations:** 1 Risk Assessment Unit, Finnish Food Safety Authority Evira, Mustialankatu 3, FI-00790 Helsinki, Finland; 2 MTT Economic Research, Latokartanonkaari 9, FI-00790 Helsinki, Finland; 3 Sjundeå Veterinärer Ab, Aleksis Kivivägen 2, 02580 Sjundeå, Finland

**Keywords:** Biosecurity, Poultry, On-farm costs, Infectious disease, Prevention, Broiler production

## Abstract

**Background:**

Farm-level biosecurity provides the foundation for biosecurity along the entire production chain. Many risk management practices are constantly in place, regardless of whether there is a disease outbreak or not. Nonetheless, the farm-level costs of preventive biosecurity have rarely been assessed. We examined the costs incurred by preventive biosecurity for Finnish poultry farms.

**Methods:**

We used a semi-structured phone interview and obtained results from 17 broiler producers and from 5 hatching egg producers, corresponding to about 10% of all producers in Finland.

**Results:**

Our results indicate that the average cost of biosecurity is some 3.55 eurocent per bird for broiler producers (0.10 eurocent per bird per rearing day) and 75.7 eurocent per bird for hatching egg producers (0.27 eurocent per bird per rearing day). For a batch of 75,000 broilers, the total cost would be €2,700. The total costs per bird are dependent on the annual number of birds: the higher the number of birds, the lower the cost per bird. This impact is primarily due to decreasing labour costs rather than direct monetary costs. Larger farms seem to utilise less labour per bird for biosecurity actions. There are also differences relating to the processor with which the producer is associated, as well as to the gender of the producer, with female producers investing more in biosecurity. Bird density was found to be positively related to the labour costs of biosecurity. This suggests that when the bird density is higher, greater labour resources need to be invested in their health and welfare and hence disease prevention. The use of coccidiostats as a preventive measure to control coccidiosis was found to have the largest cost variance between the producers, contributing to the direct costs.

**Conclusions:**

The redesign of cost-sharing in animal diseases is currently ongoing in the European Union. Before we can assert how the risk should be shared or resort to the 'polluter pays' principle, we need to understand how the costs are currently distributed. The ongoing study contributes towards understanding these issues. The next challenge is to link the costs of preventive biosecurity to the benefits thus acquired.

## Introduction

Biosecurity can be defined as the exclusion, eradication and effective management of risks posed by pests and diseases to the economy, environment and human health [1]. Risk management of biological hazards such as pests, pathogens and diseases can be broadly divided into actions which: i) take place before the biological hazard has materialised (preventive measures); ii) take place during an outbreak (eradication or control); and iii) aim at reducing the consequences in the presence of the hazard (control or adaptation). However, biosecurity is a weakest link public good, where the total amount of protection approximately equals the level of the weakest provider [2,3]. It matters little that everyone else in the production chain undertakes high biosecurity, if one of the key agents ('the weakest link') does little to prevent the entry of diseases. Hence, incentives for high biosecurity in production systems should be built into appropriate policies. The European Union (EU) has formulated the new Common Animal Health Policy 2013, with the slogan "prevention is better than cure" [4]. This promotes a shift in policy from eradication to prevention.

Although a few studies have assessed preventive actions against alternative strategies (reviewed in [5]), the farm-level costs of preventive biosecurity have rarely been assessed. A good biosecurity status requires investments in prevention. In order to assess the efficiency of biosecurity as a whole, we also need to account for the costs which ensue at those times when there are no disease outbreaks. A number of recent studies have identified key on-farm biosecurity measures in the production of beef [6], pork [7,8] and poultry [9,10]. There have also been studies assessing the benefits of preventive actions in general [11], as well as studies on farm-level economics related to optimal control of animal diseases [12,13]. However, the farm-level costs of preventive biosecurity measures have generally not been assessed. An exception is Sheppard [14], whose primary interest, however, was in assessing the total cost components in broiler production and, hence, included only vaccination and medication costs. There are also three studies from the United States dating back two decades. Sischo et al. [15] investigated the costs of preventive biosecurity in 43 dairy herds in California and found the cost of disease prevention to be US$10.72 per cow-year. Miller and Dorn [16,17] undertook a similar exercise in Ohio swine and dairy production and found preventive costs of US$6.91 per pig-year for 13 swine producers and of US$36.69 per cow-year for 16 dairy operations.

The farm-level costs are important for at least four reasons. First, many management practices are in place even in the absence of an outbreak, but an outbreak may increase these costs. Costly preventive actions may reduce the resources required and the costs of eradication in the event of a disease outbreak, although this impact has rarely been studied. Second, farm-level biosecurity provides the foundation for biosecurity in the entire production chain. This is particularly important in a system such as the EU, where the production strategy is based on biosecurity and safety in the entire production chain. Third, the farm-level costs in part determine the incentives which producers have in providing biosecurity, which is to a large extent a weakest (or weaker) link public good^a ^[3]. Identifying these costs can help in designing incentive schemes for better biosecurity. Finally, the EU is currently looking into several cost-sharing schemes related to animal diseases [18]. One factor which should be taken into account in cost sharing is the current level of expenses incurred for the different parties, including producers. Therefore, the distribution of costs and benefits in animal disease outbreaks and policies is an important issue for further study, as highlighted by the OECD [19].

We contribute towards filling the information gap regarding the current costs of biosecurity at farm level by examining the costs incurred by preventive biosecurity for Finnish poultry farms. Our aims were: 1) to estimate the total level of monetary and labour costs of preventive biosecurity in poultry farms, 2) to identify the largest and smallest cost components within the farms and 3) to obtain information regarding the variance of producer investments in specific components of biosecurity and to examine what explains this variation. The information obtained could, for instance, be used in designing cost-efficient risk management measures.

## Methods

The situation regarding poultry diseases in general is relatively good in Finland compared to many other countries in Europe and worldwide [20,21]. All parts of the food chain, including feed, the animal and food industry as well as the government, are reasonably committed to the policy of preventive biosecurity. The prevalence of *Salmonella *in Finnish poultry is normally below 1%, and as for other poultry diseases, in 2009 there were five cases of Marek's disease, and no cases of Gumboro, avian influenza or Newcastle disease [21]. The overall mortality rates in broiler production are also low.

This study ran from February to December 2007 and involved interviews with 22 poultry farms (17 broiler producers and five hatching egg producers). All the producers participated in the study voluntarily. They were selected with the assistance of Finnish poultry processors and their veterinarians who provided the contact details as well as gave assistance in persuading the producers to participate. The production of poultry in Finland is vertically highly integrated, meaning that each farm is associated with a particular poultry processor. They have a comprehensive agreement regarding the details of production, including some aspects of biosecurity, but these contracts are not publicly available. As a result, the processor has a relatively large influence on the actions of the producer. In the study, the proportion of the farms for each of the three processors is consistent with their market share.

The data were acquired through a telephone survey of the farms and concerned the 2006 production year. The questionnaire was designed with the extensive help and consultation of experts in poultry production, and it dealt with different types of actions related to biosecurity at the farm level. Only actions primarily taken for disease management purposes were included in the questionnaire. The questions were divided into 14 categories, presented in Table 1.

**Table 1:** Questionnaire categories and examples.

Category number and name	Examples
1. Biosecurity plan	Adviser fees or working hours for designing and updating written disease protection plan

2. Preventive medication	Cost of preventive medication, including the use of coccidiostats as a preventive measure to control coccidiosis and the use of a competitive exclusion product (Broilact^®^) for the prevention of intestinal disturbances in newly hatched chicks; also includes vaccination

3. Pest control	Working hours or purchased pest protection (birds, rodents, insects), pesticides, traps, scarecrows, ventilation safety nets, etc.

4. Equipment	Cost (or working hours) of machines, appliances and equipment and their maintenance concerning disease prevention, e.g. protective clothing, surface water handling

5. Education	Working hours spent on education, maintenance and updating professional skills concerning disease protection

6. Additional cleaning	Measures in addition to normal washing between the lots for disease prevention purposes (e.g. gassing)

7. Contracts for purchases and sales	Cost (or working hours) of purchase contracts for disease prevention purposes (e.g. for feed) and working hours spent on clarifying and certifying the health of the flock when selling

8. Construction plans, investments and subsidies	Working hours spent on considering disease protection when planning constructions, costs of investments in connection with disease prevention; subsidies received

9. Health monitoring programmes	Costs and working hours associated with different kinds of existing health care programmes or agreements

10. Operational hygiene	Time spent in showering before entering the production premises, as well as the time when the producer was out of the production facilitiesdue to the quarantine after a trip abroad; costs of workers' *Salmonella *certificate

11. Time period for keeping production premises empty	Possible prolonged period during which the premises are held empty for disease prevention purposes

12. Production monitoring	Costs of care for sick animals, laboratory costs, notifications and book-keeping on animal diseases and care

13. Insurance	Annual insurance fees against animal diseases

14. Control and inspections	Working hours and fees related to external inspection of animal health (by slaughter-house veterinarians, municipal veterinarians, etc.)

The questionnaire (available upon request from the authors) was sent to the producers roughly one week in advance of the actual interview call. Personal interviews were required in order to present the complex issues coherently to the producers, as well as to avoid double counting of different types of costs [22]. The interview was semi-structured: all the producers answered the same set of questions, but their answers were not restricted in any way. Further questions were asked to check the consistency of the answers. For instance, if the producer indicated a certain component costing €100 per month, it was mentioned that this would mean an annual cost of €1,200, and the producer was asked whether this was correct.

The answers given by the producers were in either euro (for direct costs or purchased services) or in hours of labour. A summary of the data is presented in Table 2. In relation to the 48-hour requirement to stay away from the poultry premises after a trip abroad (included in Category 10, operational hygiene), only 50% of the hours were taken into account. One outlier observation regarding the bird density of a farm was excluded from the calculations.

**Table 2:** Bird densities and production data

	Broiler producers				Hatching egg producers	
	**Birds/ ** **year**	**Density** **(m^2^/bird) **	**Birds/** **batch**	**Batches/** **year**	**Birds/** **year**	**Density** **(m^2^/bird) **

Mean	330,053	0.05	52,447	6.4	12,900	0.18

Median	315,000	0.05	45,000	6.5	14,800	0.19

Minimum	90,000	0.04	15,000	5.5	5,400	0.16

Maximum	774,000	0.05	129,000	7.0	18,000	0.19

The biosecurity costs were calculated for each of the categories using Microsoft Excel (Microsoft Corporation, Redmond). The costs were divided into direct monetary costs and costs in terms of labour. These were also transformed into costs per bird per year, where the annual number of birds produced was used.

In addition, we undertook ordinary least squares regression analysis with SPSS statistical software (SPSS Inc., Chicago) to examine how the costs are related to the unit size and which factors are primarily responsible for the cost variation between the farms. On the basis of background data on the correspondents, we postulated the following relationship to explain the variation in costs:

(1) $$Cost/bird_{i}=\alpha +\beta_{1}birds_{i}+\beta_{2}processorB_{i}+\beta_{3}processorC_{i}+\beta_{4}female_{i}+\varepsilon_{i}$$

The variation in the total cost per bird was assumed to depend on the size of the production unit *i *measured in terms of the number of birds produced annually (*birds_i_*) as well as on the processor with whom the producer has a contract. There were three processors in our sample, and we included dummy variables for two of them, treating the third processor as the reference level. We also postulated that the gender of the producer may matter and denoted female producers with a dummy variable. The last term *ε_i _*is the error term.

Three alternatives for the dependent variable were considered: 1) the total cost per bird, 2) the direct monetary cost per bird and 3) the labour cost per bird. The total cost is the sum of the direct monetary cost and the labour cost. Work costs were measured in hours of labour and converted into euro using an hourly wage rate of €12 per hour, the figure used in the Farm Accountancy Data Network (FADN). Additionally, in one analysis, the density of birds was used as an explanatory variable instead of the number of birds. Regression analysis was only performed for the broiler producers, as the small sample size for the hatching egg producers did not allow statistical testing.

## Results

The average total cost of preventive biosecurity for the broiler producers in our sample population was 3.55 eurocent per bird (90% confidence interval 2.56-4.40 eurocent per bird). For hatching egg producers, the expenses were higher, the mean expense being 75.7 eurocent per bird (39.3-115.5 eurocent per bird). The small number of hatching egg producer holdings did not allow for reliable statistical testing. When transformed into costs per rearing day, by dividing the cost by the average number of rearing days^b^, the figures were 0.10 eurocent per bird per rearing day for broilers and 0.27 eurocent per bird per rearing day for hatching egg producers. The general results are presented in Table 3.

**Table 3:** The costs per bird for preventive biosecurity measures in Finnish poultry (the costs per bird per rearing day in brackets)

	Cost per bird (eurocent)				90% confidence interval
	**Mean **	**Median **	**Minimum **	**Maximum **	

Broiler producers	3.55 (0.099)	3.60 (0.100)	2.52 (0.070)	4.90 (0.136)	2.56-4.40 (0.071-0.122)

Hatching egg producers	75.73 (0.271)	82.98 (0.296)	37.39 (0.134)	122.11 (0.436)	39.34-115.49 (0.146-0.428)

For broiler producers, the direct monetary cost was on average 2.51 eurocent per bird (71% of costs) and the labour cost 1.04 eurocent per bird (29%). For hatching egg producers, the corresponding figures were 49.5 eurocent (65%) and 26.2 eurocent (35%). On a per bird per rearing day basis, the corresponding figures were 0.07 and 0.03 eurocent for broiler producers and 0.18 and 0.09 eurocent for hatching egg producers.

**Figure 1:**
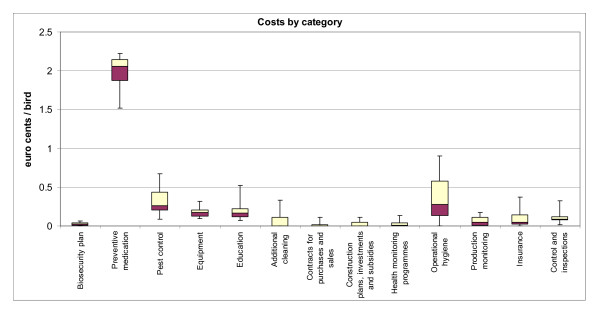
**Median on-farm biosecurity costs for broiler producers by category and per bird, together with the 25^th ^and 75^th ^(boxes) and the 5^th ^and 95^th ^(whiskers) percentiles**.

The costs were further divided into categories according to the categorisation presented in Table 1. Category 11 (time period for keeping the production premises empty) was not included in the results, as there were no cases where the production premises had been kept empty for a prolonged period of time for disease management purposes. The costs per bird in relation to the category are presented in Figure 1, where the boxes display the median as well as the 25^th ^and 75^th ^percentiles of the costs. The whiskers show the 5^th ^and 95^th ^percentiles.

The figure illustrates that only a few categories produce the majority of the expenses. The main constituent of the costs was preventive medication (Category 2), which includes the use of coccidiostats in broiler feed as a preventive measure to control coccidiosis and the use of a competitive exclusion product (Broilact^®^) for the prevention of intestinal disturbances in newly hatched chicks. The two other larger components were pest control (Category 3) and operational hygiene (Category 10).

For hatching egg producers, the equipment for biosecurity (Category 4) constituted the largest cost component, followed by preventive medication including vaccination (Category 2) and insurance against animal diseases (Category 13). The smallest cost components for both the broiler and the hatching egg producers included the biosecurity plan (Category 1), contracts for purchases and sales (Category 7), construction plans and investments (Category 8) and health monitoring programme (Category 9), the last of which can hardly be affected by the producer. Pest control (Category 3), education (Category 5) and operational hygiene (Category 10) had a similar share of total expenses for both the broiler and the hatching egg producers, each constituting about 6-10% of total biosecurity expenses.

The costs naturally varied among the producers. The largest cost variances in terms of monetary costs were found in the use of preventive medication (the difference between the highest and the lowest values of the 90% confidence interval was 0.70 eurocent), disposable requisites for pest control such as pesticides (0.25 eurocent), and insurance other than *Salmonella *(0.15 eurocent). *Salmonella *insurance and an extra level of production hygiene also stood out (both about 0.25 eurocent) and, in these cases, there were large numbers of farms with zero expense in these categories.

Similarly, for labour expenses, by far the largest cost variance (90% of variation) was related to the time taken to shower for disease prevention purposes, which varied between the farms by about 1.5 seconds, or by 0.50 eurocent, per bird. The other primary reasons for variance in labour costs were pest control, as well as the time which the producer was out of the production facilities due to quarantine (both about 1.0 seconds, or 0.30 eurocent, per bird), education, production monitoring and health monitoring programmes, and control and inspections (all three about 0.75 seconds, or 0.25 eurocent, per bird).

Regarding the impact of the unit size and other factors (equation 1), the results of the base regression using the three dependent variables are presented in Table 4. In the table, each column represents one regression, with the dependent variable at the top and the independent variables in the following rows. All three regressions were significant at the 95% confidence level, which is a good result considering that the sample size was modest. As expected, the larger the unit size (measured by the annual number of birds), the lower was the cost of biosecurity per bird. Furthermore, female producers had a higher investment in biosecurity than their male counterparts. There were also differences among the processors, with producers of processors B and C being similar, but the producers associated with processor A having a significantly lower cost of biosecurity per bird.

**Table 4:** Analysis of the factors affecting the preventive biosecurity costs on Finnishbroiler farms

	Regression 1	Regression 2	Regression 3
**Dependent variable **	**Total cost per bird **	**Direct cost per bird **	**Labour cost per bird **

Intercept	3.673 (p = 0.000)	2.417 (p = 0.000)	1.256 (p = 0.001)

Annual number of birds	-0.00000235 (p = 0.004)	-0.0000007353 (p = 0.190)	-0.00000161 (p = 0.031)

Processor B (dummy)	0.945 (p = 0.006)	0.592 (p = 0.025)	0.353 (p = 0.244)

Processor C (dummy)	0.582 (p = 0.028)	0.339 (p = 0.100)	0.243 (p = 0.326)

Female producer	0.528 (p = 0.026)	0.090 (p = 0.606)	0.438 (p = 0.060)

Regression statistics	F = 11.437 Sig. = 0.000 R^2 ^= 0.792	F = 2.602 Sig. = 0.089 R^2 ^= 0.465	F = 5.028 Sig. = 0.013 R^2 ^= 0.626

When bird density was substituted for bird number in the regression, both processor dummies became positive and significant at the 95% level. The bird density variable itself was positive and significant at the 95% level. Bird density had no statistical impact on the direct monetary costs of biosecurity (p = 0.797), but it had a statistically significant impact on biosecurity labour costs (p = 0.039) as well as on the total cost (p = 0.027). As the sign of the coefficient is positive, it suggests that one more bird per square metre increases the labour cost of biosecurity per bird by about 0.20 eurocent.

## Discussion

This study represents one of the first attempts to determine the total farm-level costs of biosecurity during a disease-free period. Our results indicate that the average cost of biosecurity is some 3.55 eurocent per bird for broiler producers (0.10 eurocent per bird per rearing day) and 75.7 eurocent per bird for hatching egg producers (0.27 eurocent per bird per rearing day). For a batch of 75,000 broilers, the total cost would be €2,700. This represents some two per cent of the total production costs and is similar in magnitude to the cost of logistics (loading and transportation) (unpublished information). The results also indicate that the work time devoted to biosecurity represents some 8% of the total work time on broiler farms and about 5% on broiler breeder farms. The costs are in the same range as the cost of vaccines and other veterinary services in England, where they were found to amount to 1.2% of total expenses and to 1.4 pence (about 1.9 eurocent) per bird [14]. The results are also qualitatively in line with those of studies undertaken on cows and pigs in the United States, where in all cases it was found that the cost of medication and biologics were the primary constituents of the disease prevention costs [15-17].

The higher cost of biosecurity per bird for the hatching egg producers is logical, since the birds spend a much longer period of time in the production facilities. However, also the cost per bird per rearing day was about three times higher for the hatching egg producers. This may suggest that the birds are more valuable to the producer and hence worth investing more. We have not compared the costs of biosecurity to the income generated by the birds in this paper. Also the higher amount of traffic in hatching egg production may explain the higher cost.

Some basic conclusions can be drawn from the regression results. First of all, regression 1 (Table 4) suggests that the total costs per bird are dependent on the annual number of birds. The sign of the coefficient is also as expected: the higher the number of birds, the lower the cost per bird. In other words, larger units incur lower costs of preventive biosecurity per bird. Second, regressions 2 and 3 reveal that this impact is primarily due to decreasing labour costs rather than direct monetary costs. Larger farms seem to utilise less labour per bird for biosecurity actions. The magnitude of the impact is such that one thousand more birds annually decrease the costs by 0.00235 eurocent. In other words, having about 425,000 more birds annually decreases the costs by one eurocent per bird (about 28%). The impact of the increased unit size on costs per bird was particularly strong in Category 14 (inspections and control).

Furthermore, it appears that the impact of processor B cannot be distinguished from that of processor C, but the farms associated with processor A (the reference level) have an approximately one eurocent lower cost of preventive biosecurity than the farms associated with the other two processors. This effect was maintained even when new variables (such as bird density) were included in the model. When examined categorically, the impact was particularly strong in Categories 2 (preventive medication), 3 (pest control) and 6 (additional cleaning). Whether this is due to more cost-effective actions, less stringent requirements of the classified production contracts or different disease pressure cannot be ascertained with this study, as we have no information on the disease history of the farms. However, since poultry production in Finland is to a large extent controlled by the processors, it is clear that the attitudes, guidelines and instructions given by the processor have an important impact at the farm level.

Female producers (24% in the sample) were also found to invest more in biosecurity. It has been observed in many studies that women are more sensitive towards risks in general [23,24] and, hence, as producers, they may also be more willing to invest in reducing the risks. The impact of a female owner was particularly visible in Categories 3 (pest control), 5 (education), 8 (construction plan, investment and subsidies) and 9 (health monitoring programmes). Unfortunately, no further background information on the producers was available.

In the dataset, bird density varied from 19 to 24 birds per m^2^. When the impact of bird density on the costs was examined, it was found that bird density was positively related to the labour costs of biosecurity. This suggests that when the bird density is higher, greater labour resources need to be invested in their health and welfare and hence disease prevention. This may be due, for instance, to the fact that the potential disease pressure is higher when the bird density is high.

In the analysis, the use of coccidiostats was found to have the largest cost variance between the producers, contributing to direct costs. The reason for this remains unclear, as the separate regressions undertaken for this cost component could not reliably relate it to any of the explanatory factors. The answer may have to do with the disease history of the farm, but that information was not available for this study.

The data for this study were collected by semi-structured interviews, which were all carried out by the same person in order to ensure inter-farm comparability. We believe that personal interviews, despite being time-consuming, are the best way to gather relatively complex data on disease prevention in a comparable way. A written questionnaire could result in more responses and thus more data to test, but the quality of the data would suffer, hence resulting in problems with statistical testing. As a result of this type of data acquisition, the amount of data collected cannot be extremely large. However, our dataset covers about 10% of Finnish producers for both production types (broiler and hatching egg). We also believe that the quality of the data more than compensates for the modest quantity.

Although the main objective in the current study was to examine the magnitude and constituents of preventive biosecurity at the level of poultry production farms, we also had some methodological issues in mind. In the future, the current methodology and the questionnaire could be applied: 1) to investigate the costs at other points in the poultry production chain as well as to determine how much the chain as a whole is investing in biosecurity and 2) to explore the costs for different types of animal production chain, including the production of pork and beef. However, for application in other countries, some modifications need to be made. Poultry production legislation and practices vary somewhat within the EU, and national regulations may differ. Hence, the questionnaire used in this study may require some revision to be applicable elsewhere.

The average size of the farms in the sample is somewhat larger than the average size of all broiler farms in Finland. However, as the trend is towards increasing farm sizes, we believe that the results are reasonably close to the current situation and are representative of the situation which we will shortly be facing. The results also suggest that if increasing farm sizes also lead to increased bird density, then greater resources will need to be invested in preventive biosecurity.

The obvious next question is whether the incurred costs effectively prevent the introduction of diseases. Furthermore, we need to know how the unit size affects the optimal level of investment on a per bird basis on preventive biosecurity. These questions cannot be answered by the results presented here. However, identifying the costs associated with preventive biosecurity is a necessary first step in understanding that biosecurity is not a free lunch.

## Conclusion

The redesign of cost-sharing in animal diseases is currently ongoing in the EU. Before we can assert how the risk should be shared or resort to the 'polluter pays' principle, we need to have an idea of how the costs are currently distributed. We investigated the costs paid by the producers for preventive biosecurity, determined the largest cost components and explained the variation in these costs. Our results indicate that the average cost of biosecurity is some 3.55 eurocent per bird for broiler producers and 75.7 eurocent per bird for hatching egg producers. For broiler producers the main constituents of the costs were preventive medication, pest control and operational hygiene. The total costs per bird are dependent on the annual number of birds: the higher the number of birds, the lower the cost per bird. This impact is primarily due to decreasing labour costs rather than direct monetary costs.

## Competing interests

The authors declare that they have no competing interests.

## Authors' contributions

KMS designed the questionnaire, undertook the interviews, processed and analysed the data, and drafted the manuscript. JH helped in designing the questionnaire, analysed the data and drafted the manuscript. NT helped in designing the questionnaire and in drafting the manuscript. HR initiated the study, helped in designing the questionnaire and in analysing the data, and drafted the manuscript. All authors read and approved the final manuscript.

## Endnotes

a) Biosecurity is a public good because it is non-excludable in production and non-rival in consumption. Non-excludability means that once protection against the disease is provided, no agent (e.g. no farm) can be excluded from enjoying the benefits. For instance, if disease entry is prevented, nobody can be prevented from enjoying that. Non-rivalry means that one agent's consumption of protection does not reduce the amount of protection enjoyed by others. These are the two elements of a pure public good. Moreover, biosecurity is often of the 'weakest link' type, because its effectiveness depends on the weakest link in the protection chain. It does not matter how well other parts of the chain provide protection if the disease gets into the country through the weakest control point. A milder version of this argument (a weaker link) can be found in [2]. In this case, the investment of those who invest more on protection is negatively affected by those who invest less, but those who invest more are still better protected than those who invest less.

b) The rearing times in Finland at the time of this study were approximately 33-39 days for broilers and about 40 weeks, i.e. 280 days, for the parents (personal communication, veterinarian of a Finnish poultry processor).
